# Genus-level phylogeny of cephalopods using molecular markers: current status and problematic areas

**DOI:** 10.7717/peerj.4331

**Published:** 2018-02-12

**Authors:** Gustavo Sanchez, Davin H.E. Setiamarga, Surangkana Tuanapaya, Kittichai Tongtherm, Inger E. Winkelmann, Hannah Schmidbaur, Tetsuya Umino, Caroline Albertin, Louise Allcock, Catalina Perales-Raya, Ian Gleadall, Jan M. Strugnell, Oleg Simakov, Jaruwat Nabhitabhata

**Affiliations:** 1 Graduate School of Biosphere Science, Hiroshima University, Higashi-Hiroshima, Hiroshima, Japan; 2 Molecular Genetics Unit, Okinawa Institute of Science and Technology, Okinawa, Japan; 3 Department of Applied Chemistry and Biochemistry, National Institute of Technology—Wakayama College, Gobo City, Wakayama, Japan; 4 The University Museum, The University of Tokyo, Tokyo, Japan; 5 Department of Biology, Prince of Songkla University, Songkhla, Thailand; 6 Section for Evolutionary Genomics, Natural History Museum of Denmark, University of Copenhagen, Copenhagen, Denmark; 7 Department of Molecular Evolution and Development, University of Vienna, Vienna, Austria; 8 Department of Organismal Biology and Anatomy, University of Chicago, Chicago, IL, United States of America; 9 Department of Zoology, Martin Ryan Marine Science Institute, National University of Ireland, Galway, Ireland; 10 Centro Oceanográfico de Canarias, Instituto Español de Oceanografía, Santa Cruz de Tenerife, Spain; 11 Graduate School of Agricultural Science, Tohoku University, Sendai, Tohoku, Japan; 12 Marine Biology & Aquaculture, James Cook University, Townsville, Queensland, Australia; 13 Excellence Centre for Biodiversity of Peninsular Thailand, Prince of Songkla University, Songkhla, Thailand

**Keywords:** Cephalopods, Phylogeny, Molecular markers

## Abstract

Comprising more than 800 extant species, the class Cephalopoda (octopuses, squid, cuttlefish, and nautiluses) is a fascinating group of marine conchiferan mollusks. Recently, the first cephalopod genome (of *Octopus bimaculoides*) was published, providing a genomic framework, which will enable more detailed investigations of cephalopod characteristics, including developmental, morphological, and behavioural traits. Meanwhile, a robust phylogeny of the members of the subclass Coleoidea (octopuses, squid, cuttlefishes) is crucial for comparative and evolutionary studies aiming to investigate the group’s traits and innovations, but such a phylogeny has proven very challenging to obtain. Here, we present the results of phylogenetic inference at the genus level using mitochondrial and nuclear marker sequences available from public databases. Topologies are presented which show support for (1) the monophyly of the two main superorders, Octobrachia and Decabrachia, and (2) some of the interrelationships at the family level. We have mapped morphological characters onto the tree and conducted molecular dating analyses, obtaining congruent results with previous estimates of divergence in major lineages. Our study also identifies unresolved phylogenetic relationships within the cephalopod phylogeny and insufficient taxonomic sampling among squids excluding the Loliginidae in the Decabrachia and within the Order Cirromorphida in the Octobrachia. Genomic and transcriptomic resources should enable resolution of these issues in the relatively near future. We provide our alignment as an open access resource, to allow other researchers to reconstruct phylogenetic trees upon this work in the future.

## Introduction

Animals of the Class Cephalopoda (octopuses, squid, cuttlefishes, and nautiluses) inhabit a wide range of marine environments, from the tropical to the polar regions and from neritic to oceanic zones. The class contains more than 800 described species, which exhibit a wide range of body sizes (∼1 cm to ∼3 m dorsal mantle length), and highly diverse morphologies (Jereb & Roper, 2010), life styles, and behaviours (Hanlon & Messenger, 1998). Cephalopods are an important food source in many parts of the world, and thus they are an important target for commercial fisheries.

Although a consensus remains elusive for stable resolution of phylogenetic relationship among the major molluscan classes (Stöger et al., 2013), the phylogenetic position of the Cephalopoda among other molluscan lineages has been demonstrated by three independent phylogenomic studies of molluscs (Kocot et al., 2011; Smith et al., 2011; Telford & Budd, 2011). Along with the classes Gastropoda (snails and limpets), Bivalvia (oysters and mussels), Scaphopoda (tusk shells) and Monoplacophora, the Cephalopoda are included among the conchiferan molluscs (Kocot et al., 2011; Smith et al., 2011), which have in common an external, calcified shell.

Extant cephalopods are classified into two distinct groups which diverged long ago: the ancient, shelled nautiloids (Subclass Nautiloidea) and the rapidly evolving Subclass Coleoidea. The latter includes two major groups: Octobrachia (octopuses); and Decabrachia (squids and cuttlefishes). Unlike the nautiloids, extant coleoid cephalopods have no external calcified shell. Some, such as the Ram’s horn squid (Family Spirulidae) and the cuttlefishes (Family Sepiidae), have an internal calcified shell or phragmocone; whilst in the remaining decabrachian groups the shell has been reduced to a gladius or lost completely. In the Octobrachia, there may be shell remnants in the form of paired stylets, a cartilaginous fin support, or it may be completely absent.

Interrelationships among extant Cephalopoda have been difficult to resolve. Strong support has been shown for monophyly of the major extant groups (the subclasses Nautiloidea and Coleoidea, and the two major coleoid superorders, Decabrachia and Octobrachia), but phylogenetic resolution among lower ranks within these groups remains unclear (Allcock, Lindgren & Strugnell, 2014).

Molecular phylogenetic analysis has been employed in attempts to resolve the phylogenetic relationships at various taxonomic levels within the Subclass Coleoidea (reviewed recently by Allcock, Lindgren & Strugnell, 2014). Several genetic markers have been used, both nuclear (i.e., 18S rRNA, histone H3, octopine dehydrogenase (ODH), pax-6, rhodopsin, and actin) and mitochondrial (i.e., cytochrome c oxidase subunit I (COI), 12S rRNA, and 16S rRNA). Studies using mitogenomes also have been conducted, based not only on amino acid and nucleic acid sequences (Allcock, Cooke & Strugnell, 2011; Bonnaud, Boucher-Rodoni & Monnerot, 1997; Cheng et al., 2013), but also on gene order rearrangements within the genomes (Akasaki et al., 2006; Allcock, Cooke & Strugnell, 2011; Strugnell et al., 2017; Uribe & Zardoya, 2017).

In the present study, all publicly available molecular markers were used, both nuclear and mitochondrial, in an attempt to investigate the phylogenetic relationships within the Cephalopoda. The aim is to create an overview of which groups are proving the most difficult to resolve, and which groups are already robustly supported, thus providing a general roadmap for future systematic studies. The results and matrix presented here are a further step towards resolving the less clear relationships among members of Cephalopoda and should form a base upon which future studies using large data sets can be built, such as comparative genomics and phylogenomics.

## Materials & Methods

### Taxon sampling and sequence acquisitions

All publicly available molecular markers (nuclear: 18S rRNA, *Histone H3*, *octopine dehydrogenase*, *pax-6*, *rhodopsin*, *actin*; mitochondrial: cytochrome c oxidase subunit I [COI], 12S rRNA, 16S rRNA) and all the 18 available full mitochondrial genomes of cephalopods available as of November 2015 were retrieved from GenBank and the Barcode of Life Database (Ratnasingham & Hebert, 2007). All available species within a genus were used to create a genus-level dataset. For those genera for which multiple species or multiple sequences were available, we selected only the entry with the longest sequence (Table S1). We referred to the latest taxonomic information (Jereb & Roper, 2010; Strugnell et al., 2014) and corrected those sequences for which taxonomic data had been misidentified or misplaced (Table S2). A second filtering step was conducted by discarding all sequences that had previously been reported as contaminants (Lindgren et al., 2012).

### Sequence checks, alignments, and editing

Sequences were aligned using MAFFT v7.158b (Katoh et al., 2002) with default settings. The alignments were then processed with Gblocks 0.91b (Castresana, 2000) to eliminate poorly aligned positions from the final matrix. To increase the number of phylogenetically informative variable sites, gaps were allowed in up to half of the genera, as determined by Gblocks.

Preliminary rounds of phylogenetic inference using a maximum likelihood analysis in RAxML v.8.2.4 (Stamatakis, Ludwig & Meier, 2005) enabled us to identify and remove contaminants and poorly aligned sequences, by inspecting both abnormal branch lengths and alignment quality. When contaminants were detected, all markers linked with that particular genus were removed from the analysis.

**Figure 1:**
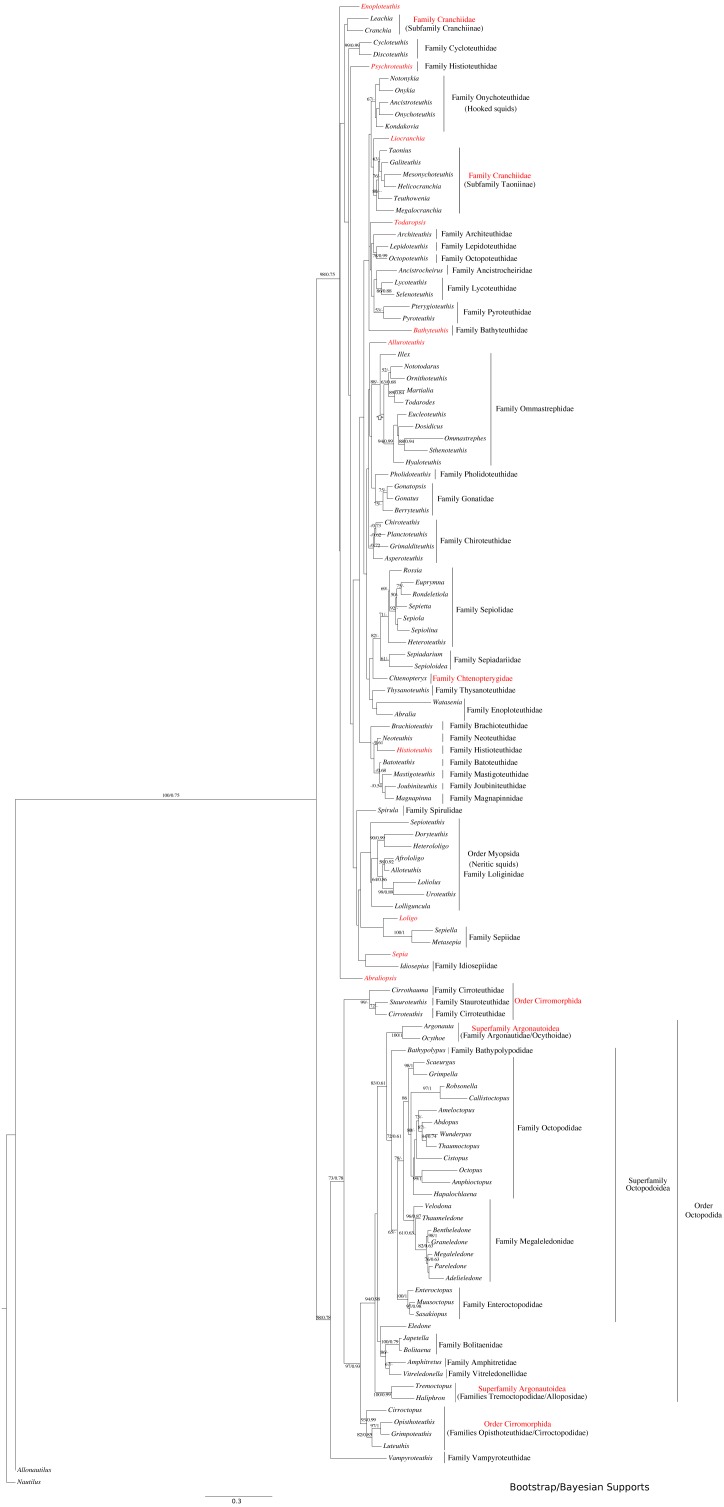
Maximum-likelihood and Bayesian supported tree of the Cephalopoda. Taxa highlighted in red represents discrepancy to previously published studies.

**Figure 2:**
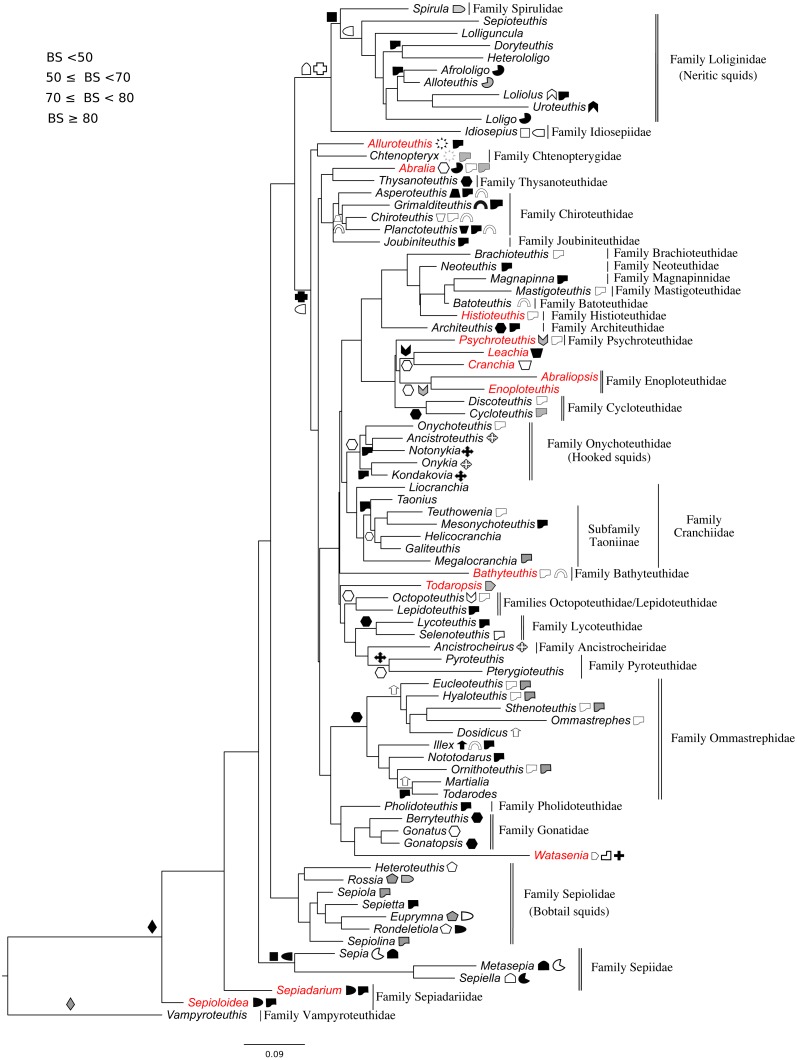
Maximum-likelihood tree of the Decabrachia under the GTR + Gamma model with the morphological character set mapped onto the tree. Taxa highlighted in red represents discrepancy to previously published studies.

**Figure 3:**
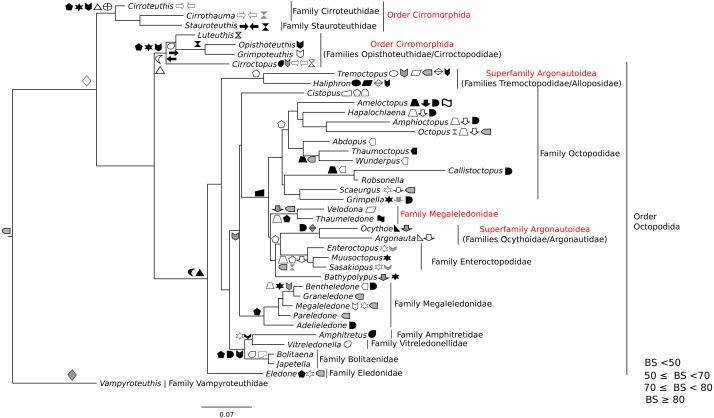
Maximum-likelihood tree of the Octobrachia under the GTR + Gamma model with the morphological character set mapped onto the tree. Taxa highlighted in red represents discrepancy to previously published studies.

All markers were aligned individually, then concatenated to produce the final alignment. If any conflict was detected (low alignment quality or branch length discrepancy), marker sequences were filtered and aligned again. Three sequence alignments and phylogenetic trees were created: (1) Subclass Coleoidea (using *Nautilus* of the Subclass Nautiloidea as the outgroup, Fig. 1), (2) Superorder Decabrachia (Fig. 2), and (3) Superorder Octobrachia (Fig. 3). Aligned and trimmed sequence data sets are available at https://doi.org/10.6084/m9.figshare.5116759.

### Phylogenetic inference

Partitioned maximum likelihood (ML) analysis was performed using RAxML v.8.2.4 on the computational cluster at the Okinawa Institute of Science and Technology (OIST) with 1,000 bootstrap replicates under the GTR model of evolution with Gamma distribution (GTR+Gamma). *Nautilus* was used as the outgroup for the analysis of all cephalopods, and *Vampyroteuthis* was used as the outgroup for analyses of both the Decabrachia and Octobrachia. The concatenated dataset was partitioned by gene for the phylogenetic tree reconstruction.

In addition (again using *Nautilus* as the outgroup), Bayesian inference of all cephalopods was conducted in MrBayes-MPI v.3.2.2 (Huelsenbeck & Ronquist, 2001) with different models as inferred in jModelTest 2.1.10 (Darriba et al., 2012) using the Bayesian information criterion. Two independent runs were conducted for 20 million generations, sampling the Markov chain every 10 generations and each run having one cold and three heated chains with a STOPRULE option, halting the analysis when the average standard deviation of split frequencies reached 0.01. The first 25% of trees were removed as burn-in. Posterior probabilities of the clades with a majority-rule consensus tree were summarized. The convergence within chains was assessed by an effective sample size (ESS) of more than 500 using Tracer v1.6.0 (Rambaut et al., 2014).

Clades were considered not supported for bootstrap support (BS) values <50 %; resolved for BS between 50% and 70%; well supported for BS between 70% and 80%; and strongly supported for BS >80%.

### Morphological character mapping

A character set was created for both Decabrachia and Octobrachia (Tables 1 and 2) based on our final tree topologies and mapped onto them (Figs. 2 and 3). Morphological characters were extracted from (Jereb & Roper, 2010; Jereb & Roper, 2005; Jereb et al., 2013), based on their information in defining synapomorphies at the appropriate level (Young & Vecchione, 1996). The majority of the characters are qualitative, readily visible on inspection of fresh specimens. These character sets were dichotomously aligned to each operational taxonomic unit (OTU; generic level) on the branches of molecular trees. Symbols are used to represent the presence of characters on tree branches. Some characters are mapped only in a subset of OTUs (indicated in Tables 1 and 2); their absence from others indicates either that they are not relevant within that group, or that we could not assess them within a given clade at present. This current character set can be modified and extended further in future phylogenetic studies.

**Table 1:** Morphological character set and symbols created for the Decabrachia phylogenetic trees.

Morphological Characters of Decabrachia (18 characters)							
No.	Character	Character State					
1	Adhesive Organ on Dorsum (Idiosepiidae, Loliginidae)	Absent	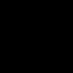	Present	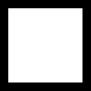		
2	Arm Length (Chiroteuthidae)	>5 times ML	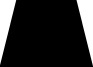	<5 times ML	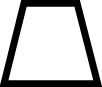		
3	Arm (Oral Appendages) Number	10	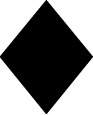	8	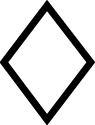	8 + 2 filamentous	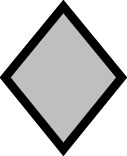
4	Arm Sucker Series^a^ (Sepiolidae)	1	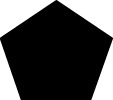	2	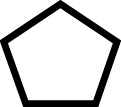	4	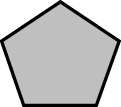
5	Arm and/or Club Hook	Absent	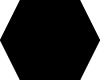	Present	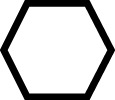		
6	Cuttlebone Spine (Sepiidae)	Absent	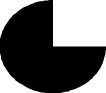	Present	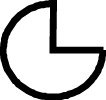		
7	Eye Cornea	Absent	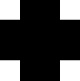	Present	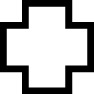		
8	Fin Ribbed (Chtenopterygidae)	Absent	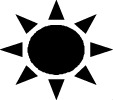	Present	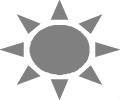		
9	Funnel Groove Foveola (Ommastrephidae)	Absent	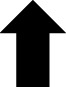	Present	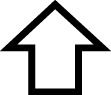		
10	Funnel Valve (Cranchiidae, Chiroteuthidae)	Absent	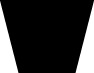	Present	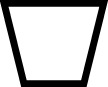		
11	Gladius Rostrum (Loliginidae)	Absent	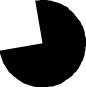	Thin	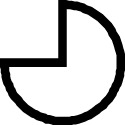		
12	Internal Shell (Sepiolidae)^a^	Absent	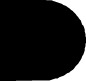	Present/ Rudiment	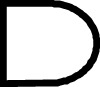	Present/ Coiled	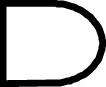
13	Internal Shell Uncoiled^a^	Single/Calcified (cuttlebone)	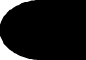	Single/Chitinous (gladius)	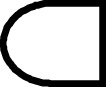		
14	Lifestyle of Hatchling	Benthic	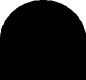	Planktonic	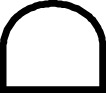		
15	Mantle Tissue^a^	Gelatinous	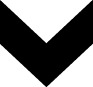	Semi-gelatinous	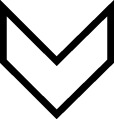	Muscular	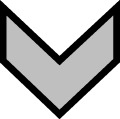
16	Nuchal Fold	Absent	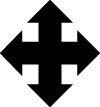	Present	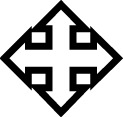		
17	Photophores^a^	Absent	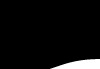	Present/External (mantle, arm)	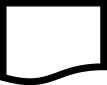	Present/ Internal (viscera)	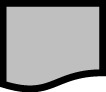
18	Tentacular Club Sucker Series	<4	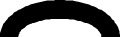	≥4	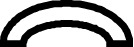		

**Notes.**

a Characters shared in both Decabrachia and Octobrachia.

**Table 2:** Morphological character set and symbols created for the Octobrachia phylogenetic trees.

Morphological Characters of Octobrachia (21 characters)									
No.	Character	Character state							
1	Arm Cirri	Absent	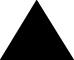	Present	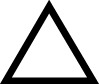				
2	Arm Length (Octopodidae, Megaleledonidae, Enteroctopodidae)	>5 times ML	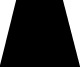	<5 times ML	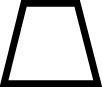				
3	Arm (Oral Appendages) Numbers^a^	8	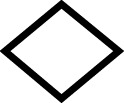	8 + 2 filamentous		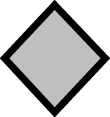			
4	Arm Sucker Series^a^	1	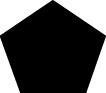	2	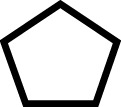	4	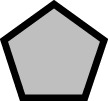		
5	Arm Web (Argonautoidea: Ocythoidae, Argonautidae)	Absent	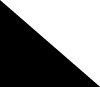	Present/ Shallow	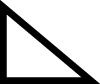	Present/ Deep	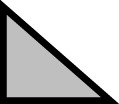		
6	Arm Web Extension (Argonautoidea: Tremoctopodidae, Alloposidae)	Absent	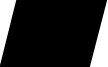	Present	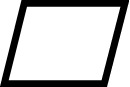				
7	Cephalic Water Pore (Argonautoidea)	Absent	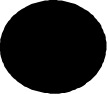	Present	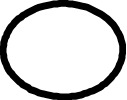				
8	Eye Position (Cirromorphida Opisthotheutidae, Cirroctopodidae, Vitreledonellidae, Amphitretidae, Bolitaenidae)	Dorsal	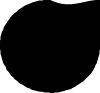	Lateral	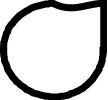				
9	Fin	Absent	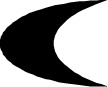	Present	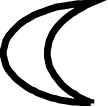				
10	Fin Width (Cirromorphida)	<100% ML	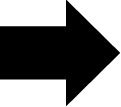	>100% ML	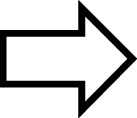				
11	Fin Length (Cirromorphida)	<50% ML	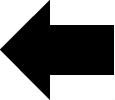	>50% ML	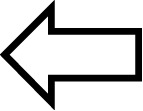				
12	Funnel Organ (Octopodidae, Argonautoidea, Ocythoidae, Argonautidae, Enteroctopodidae)	Absent/ Reduced	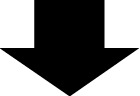	Present/W shape	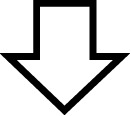	Present/V shape	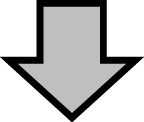	Present/ U shape	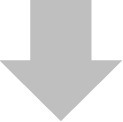
13	Hectocotylus Calamus (Octopodidae, Megaleledonidae)	Absent	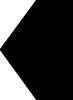	Present	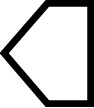				
14	Ink Sac (Cirromorphida, Enteroctopodidae, Bathypolypus, Megaleledonidae)	Absent	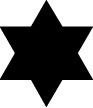	Present	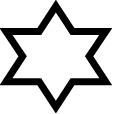				
15	Internal Shell^a^ (Octopodidae, Argonautoidea, Ocythoidae, Argonautidae, Megaleledonidae, Enteroctopodidae)	Absent	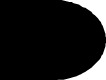	Present/ Rudiment	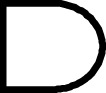	Present/ Coiled	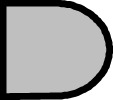	Present/ Uncoiled	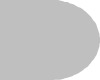
16	Internal Shell Uncoiled^a^	Single/Calcified (cuttlebone)	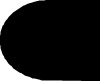	Single/ Chitinous (gladius)	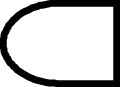	Single and Paired/Stylet	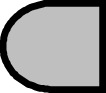		
17	Interbrachial Web Pouches (Octopodidae, Megaleledonidae, Enteroctopodidae)	Absent	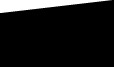	Present	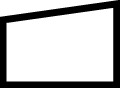				
18	Mantle Tissue^a^	Gelatinous	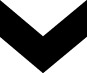	Semi-gelatinous	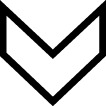	Muscular	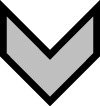		
19	Photophores^a^ (Bolitaenidae)	Absent	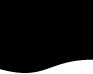	Present/ External (mantle, arm)	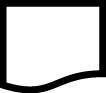	Present/Internal (viscera)	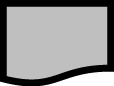		
20	Radula Teeth Component (Octopodidae, Megaleledonidae)	5	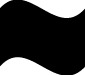	>5	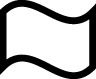				
21	Stylet Shape	U	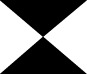	V	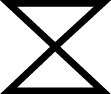	W	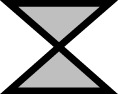	Rod/Saddle	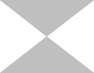

**Notes.**

a Characters shared in both Decabrachia and Octobrachia.

### Divergence date estimation

Divergence times among the major cephalopod lineages were estimated using the r8s v1.80 program (Sanderson, 2003). The time of coleoid divergence from Nautiloidea was fixed at 270 mya (Kroger, Vinther & Fuchs, 2011) and allowed for a local model with variable mutation rate index in both superorders Octobrachia and Decabrachia. Rates were estimated with the LF (Langley-Fish) model and 10 rate categories.

## Results

### Marker distribution and general tree topology

Our data includes the largest collection of molecular markers for cephalopods to date (available as of November 2015). Our focus on genus-level availability of the sequence data permitted a final concatenated data matrix of 15,713 bp, spanning 124 genera and, mitochondrial and nuclear genes representing 74% and 26% of the total matrix, respectively. 16S rRNA and COI were the most commonly available markers, representing 119 and 113 genera, respectively. Only 3,212 out of 15,713 positions had more than half of the 124 genera represented, due to the low taxonomic sampling of mitochondrial genomes.

### General results for the phylogenetic tree

The use of concatenated sequences of all markers (Fig. 2) resulted in a resolved topology for monophyly of the Octobrachia (BS = 58%), and strong support for monophyly of the Decabrachia (BS = 98%), with both clades strongly supported by the Bayesian approach with PP = 0.78 and 0.75 respectively. Although monophyly was demonstrated for several families contained within both superorders, the relationships of the families contained within Octobrachia were better supported than those in Decabrachia (Fig. 2). Of the 37 nodes in the Octobrachia portion of the general tree containing all taxa, the majority were resolved above the 50% level (31 nodes with BS > 50%); but only 28 out of 80 nodes in the Decabrachia were resolved at BS >50%, most of which were located at family level.

ML and Bayesian support analyses of the Octobrachia returned a strongly supported Octopodida (BS = 94%, PP = 0.98), but found the Order Cirromorphida to be paraphyletic. The Octopodida contained several strongly supported family-level clades, including the Octopodidae (BS = 86%, PP = 0.61), Amphitretidae (BS = 86%), Enteroctopodidae (BS = 100%, PP = 1), and Megaleledonidae (BS = 96%, PP = 0.87). Benthic families possessing a double row of suckers (i.e., Enteroctopodidae, Octopodidae and Bathypolypodidae) together with the Megaleledonidae (possessing a single row of suckers) formed a well-supported monophyletic group (BS = 72%, PP = 0.61). Additionally, the families Tremoctopodidae and Alloposidae together formed a strongly supported clade (BS = 100%, PP = 0.99), as did the families Argonautidae and Ocythoidae (BS = 100%, PP = 1). However, the superfamily Argonautoidea appears to be paraphyletic. Eledonidae and Amphitretidae were recovered as sister taxa, but this relationship was not resolved at the BS threshold of 50%.

Support was low for subgroups of the Decabrachia within this general tree of the Cephalopoda. Even the clades that have remained relatively stable across several previous studies, such as the Myopsida (squids with a closed eye capsule), showed no significant bootstrap support within the topology from this analysis. The current study, therefore, focussed on separate analyses of the Decabrachia and Octobrachia as independent trees.

### Phylogeny of the decabrachia

We identified 10 well or strongly supported families within the Decabrachia (BS > 71%; Fig. 2, double bars), but the relationships among these families remain uncertain due to very low bootstrap support values and short branch lengths at this phylogenetic level. Several well established taxonomic clades were recovered, including Sepiolidae (bobtail squids; BS = 85%), Loliginidae (BS = 71%) and Sepiidae (BS = 73%). Interestingly, Idiosepiidae, Spirulidae and Loliginidae formed a monophyletic group in the present study but resolved at only 57% of BS. Although the oegopsids (open-eyed squids: squids other than the Loliginidae) formed a monophyletic group in the ML topology, it was not resolved (BS < 50%). However, several groups among the Oegopsida were mostly strongly supported, including the Gonatidae (BS = 77%), Taoniinae (BS = 86%), Cycloteuthidae (BS = 99%), Lycoteuthidae (BS = 94%) and Ommastrephidae (BS = 88%; with the exception of *Todaropsis*) (Fig. 2).

This analysis yielded greater resolution for some of the problematic genera in the tree including both Decabrachia and Octobrachia together (genera or family marked with red in Figs. 1 and 2). Thus, the all-Coleoidea tree shows several apparently misplaced taxa (in particular *Abraliopsis*, *Sepia*, *Loligo*, *Alluroteuthis*, *Todaropsis*, *Liocranchia*, *Pysochroteuthis, Histioteuthis* and *Enoploteuthis*); while some oddities remained in the Decabrachia-only tree, but with lack of support for all. This includes the apparent polyphyly of members of the Families Enoploteuthidae, Cranchiidae, Bathyteuthoidea, Ommastrephidae, *Pysochroteuthis, Histioteuthis*; and the apparent basal position of the *Sepioloidea* and *Sepiadarium*. Additionally, several families that are believed to be closely related, for example Histioteuthidae-Psychroteuthidae, Lepidoteuthidae-Octopoteuthidae and Chiroteuthidae-Mastigoteuthidae-Batoteuthidae, were not recovered as monophyletic clades. These families show better support for a different phylogenetic position in previous studies (Lindgren, 2010, Lindgren et al., 2012; Tanner et al., 2017; Uribe & Zardoya, 2017).

### Phylogeny of the octobrachia

Maximum likelihood phylogenetic analysis of Octobrachia recovered a strongly supported Octopodida (BS = 98%), but the Order Cirromorphida appears paraphyletic. Two cirromorphid clades were resolved and strongly supported, one containing the families Opisthoteuthidae and Cirroctopodidae (BS = 67%), and the other Stauroteuthidae and Cirroteuthidae (BS = 90%). Several families were strongly supported within the phylogeny, including the Enteroctopodidae (BS = 99%), Octopodidae (BS = 95%) and Amphitretidae (BS = 89%). The Superfamily Argonautoidea was not resolved as monophyletic. As in the Decabrachia tree, oddities or discrepancies found in the Octobrachia are highlighted in red.

### Estimation of divergence times

The long branch lengths separating nautiloid and coleoid cephalopods support their reported late Cambrian origins (Kroger, Vinther & Fuchs, 2011). By calibrating the Octobrachia -Decabrachia divergence at 270 Mya (Kroger, Vinther & Fuchs, 2011), it is possible to estimate approximate times of divergence among the major groups. This produced estimated divergence times of ∼220 Mya between the Octobrachia and Vampyromorphida, and 150 and 170 Mya for Decabrachia and Octobrachia lineages, respectively. This is roughly consistent with recent studies, including estimates based on the fossil record (Kroger, Vinther & Fuchs, 2011), transcriptomic studies (∼212 and ∼219 Mya for Octobrachia and Decabrachia, respectively in Tanner et al., 2017), and other markers (Warnke et al., 2011). Interestingly, the divergence between the major decabrachian lineages appears to have occurred within a relatively short period of time (e.g., within a 20 million-year time frame for the Loliginidae).

## Discussion

### Congruence of molecular and morphological characters

Our trees provide interesting insights into the evolution of the major octobrachian lineages. We identify a monophyletic clade containing the benthic octopods Octopodidae, Bathypolypodidae, Enteroctopodidae and Megaledonidae. A monophyletic group containing these octopods was also recovered by Strugnell et al. (2014) from analysis of nucleotide sequences of three nuclear genes (*rhodopsin, pax-6* and *octopine dehydrogenase*). This topology could reflect the fact that the present study contains data used in that study. However, it should be noted that Strugnell et al. (2014) failed to recover this clade in analyses of nucleotide sequences of mitochondrial genes alone, from nuclear and mitochondrial genes together, or from amino acid data. Confirming earlier studies (Voight, 1993; Allcock & Piertney, 2002; Gleadall, 2004; Allcock et al., 2006; Ibáñez, Sepúlveda & Chong, 2006; Gleadall et al., 2010; Strugnell et al., 2014), we find that loss of the ink sac has occurred independently several times (e.g., in some members of the Enteroctopodidae, Megaleledonidae and Octopodidae, and in Bathypolypodidae).

The Octopodidae, Enteroctopodidae, Bathypolypodidae, and Megaleledonidae form a monophyletic clade characterised by benthic adults, while the clades diverging earlier within the Octopodida (including Amphitretidae and Argonautoidea) are pelagic (or benthopelagic in the case of *Haliphron*), with the exception of *Eledone*. This topology provides some support for the hypothesis that pelagic octopod families (Argonautoidea and Amphitetidae) may not have evolved a pelagic lifestyle independently, but could have evolved from a common pelagic ancestor (see Young, Vecchione & Donovan, 1998, for a view counter to this). Nevertheless, the weak support values for some clades highlights the importance of additional sequencing across more taxa for some genes (Table S1).

Surprisingly, the Suborder Cirromorphida appears as paraphyletic group in our results for analyses of both the all-Coleoidea and the Octobrachia alone (Figs. 1 and 3). Previously reported as monophyletic (Piertney et al., 2003) and supported by morphological characters such as the plesiomorphic ovoid sperm packets, their strongly supported split and basal position to the Octopodida are unlikely to represent a real scenario. If the present phylogeny is correct, it could imply either (1) the ancestral character, spermatophores (present in Nautiloids and most of Coleoids), were lost and the ovoid sperm packets evolved twice or (2) the ancestor of the Octobrachia had ovoid sperm packets and the octopodids lost them and evolved the conventional character, spermatophores. As these are unlikely events, we believe that this phylogeny signal could be the result of saturation of the DNA markers in the Cirromorphids, which are mainly represented 16S rRNA and COI that can be saturated in deep nodes of the Octobrachia tree.

### Phylogenetic relationships within the Decabrachia

While the major clades are supported for the Decabrachia, there are some interesting outliers. Contrary to previous studies suggesting that *Idiosepius* diverged early (e.g., Sutton, Perales-Raya & Gilbert, 2016), in the present study, it forms a clade with the Loliginidae and Spirulidae in both the coleoid-inclusive and Decabrachia-only trees. This is also contrary to the monophyletic relationship of *Idiosepius* and Sepiolida that is strongly supported by mitochondrial gene rearrangements (Strugnell et al., 2017) and transcriptomic data (Tanner et al., 2017). Our results may thus be an artefact of the genes chosen for analysis since (similar to our study), Strugnell et al. (2017) found *Idiosepius* to be an unsupported sister taxon to the Loliginidae when based on the mitochondrial genes alone.

The position of the Spirulidae as a sister to the Loliginidae was not supported in this study but it has been found to form a sister taxon to the oegopsids for both transcriptomic and mitogenomic analyses (Strugnell et al., 2017; Tanner et al., 2017).

Monophyly in the commercially exploited family Ommastrephidae is well supported. Our analyses grouped almost all ommastrephid genera, with the exception of *Todaropsis*. We recovered two major subgroups (one containing *Ommastrephes* and the other *Todarodes*), which can be distinguished by the presence of subcutaneous mantle photophores. Nevertheless, the branching pattern of the ommastrephid genera remains unsupported and highlights the importance of further gene sequencing. Phylogenetic work on most of the ommastrephid species is limited to the COI and 16S mitochondrial markers (Wakabayashi et al., 2012).

The inclusion of *Lepidoteuthis* (Lepidoteuthidae) and *Octopoteuthis* (Octopoteuthidae) as a monophyletic taxon is in accordance with previous studies based on COI and morphological characters (Carlini & Graves, 1999; O’Shea, Jackson & Bolstad, 2007; Sutton, Perales-Raya & Gilbert, 2016).

Our results showed no support for most of the deep branches of the Decabrachia. However, some relationships have been addressed in recent mitogenome (Strugnell et al., 2017) and transcriptomic studies (Tanner et al., 2017), where these unsupported clades were recovered as strongly supported. Contrary to our results, Tanner et al. (2017) found that the Sepiolida forms a well–supported sister group together with the Teuthoidea including *Spirula*. Moreover, these other studies together with Sutton, Perales-Raya & Gilbert (2016) (based on morphological data from living and fossil taxa) placed the benthic cuttlefish species (Family Sepiidae) as a basal clade of the Decabrachia, which may be a hint that the ancestral decabrachian lifestyle was benthic (Young, Vecchione & Donovan, 1998). This is in contrast to the pelagic-to-benthic transition proposed for the Octobrachia.

### Divergence time estimation and the Decabrachia conundrum

Our results for times of divergence reveal a relatively closely-spaced radiation across the decabrachian lineages. The general pattern of low levels of bootstrap support and short branch lengths could therefore be a consequence of rapid radiation of the major decabrachian groups before the late Jurassic period (based on the estimate of 150 mya). Roughly similar results have been reported by Tanner et al. (2017) based on transcriptomic data, suggesting that this rapid radiation happened during the middle of the Jurassic period.

### Methodological shortcomings and future directions

Methods that rely heavily on mitochondrial markers are vulnerable to sequence bias and saturation affecting the tree topology (Springer et al., 2001). Despite being largely congruent with previous analyses, our data are lacking in nuclear marker coverage. Current efforts, especially using transcriptomics (Kocot et al., 2011; Tanner et al., 2017), will allow for a much larger sampling of thousands of orthologous genes, that couple with the current morphological character set provided, will yield a better insight into many taxa evolutionary relationship.

Since it will likely be a huge undertaking to thoroughly sequence all the genes for which data are currently lacking, this study makes it possible to recognise specific areas of the tree upon which more attention would be fruitful for consideration in future transcriptome or mitogenome studies. Recent work using mitogenomes (Strugnell et al., 2017) and transcriptomes (Tanner et al., 2017) have already established the relationships of major clades such as, octopodids, loliginids, sepiids, sepiolids and oegopsids, but it is clear that further taxon sampling is necessary for the Oegopsida, which includes about 230 species in more than 20 families (Sweeney & Roper, 1998).

Another relationship requiring further attention is the Subfamily Sepiolinae (*Euprymna* and *Sepiola*), for which clear key morphological features are almost restricted to the hectocotylus shape and enlarged suckers in males, and with a large number of species reported in the Mediterranean Sea, South East Asia and Japan. Misidentification of species is apparently high for this group (see, for example, Groenenberg et al., 2009) and the current number of DNA markers is limited. In addition, the relationship of the Sepiolinae with the other members of the Sepiolidae remains unresolved. More sequencing or transcriptomic data of the clades mentioned above should contribute to improved understanding of their inter-relationships and yield more accurate divergence time estimates.

The topology within the Octobrachia, while stable, would benefit greatly from obtaining transcriptomic sequences for the clades that apparently diverged earliest, such as Cirromorphida. Additional efforts should also be made into the relationship of the cirromorphids with the remaining taxa of the Octopodida, as the result present in this study indicates a potential saturation due to the high number of mitochondrial compared with nuclear markers. Further data should also contribute to a better understanding of the relationship between *Vampyroteuthis* and the Octobrachia and Decabrachia. Combined with the data available from the *Octopus bimaculoides* genome project (Albertin et al., 2015), transcriptomes of a megaleledonid and an enteroctopodid would help resolve the evolutionary history of morphological traits, such as double versus single sucker columns within the benthic clades. Additional groups of interests to investigate in more detail would be within the Argonautoidea, the remaining Octopodida and the Cirromorphida (∼250 species; Sweeney & Roper, 1998).

## Conclusions

To our knowledge, the analysis presented here includes the largest taxonomic coverage and collection of marker sequences for the Cephalopoda to date. The results provide insight into the robustness of the topologies across the global cephalopod phylogenetic tree, as well as the resolution of interrelationships at both family and genus levels. Additionally, our analyses reveal the need for further taxon sampling, more DNA markers, mitogenomic and transcriptomic data for several cephalopod clades, especially within the oegopsids and Cirromorphida. Future studies might take advantage of the increasing ease with which dense genome-wide data sets can be generated via high-throughput sequencing. Although some have argued that further resolution may not necessarily be forthcoming (see, for example, Philippe et al., 2011), phylogenomics and/or comparative genomics grounded in such big-data-based analyses have the potential to resolve phylogenetic relationships among the cephalopods, and particularly to investigate further the phylogenetic interrelationships of the high supported subclades within the Decabrachia and Octobrachia.

##  Supplemental Information

10.7717/peerj.4331/supp-1 Supplemental Information 1 Table S1 and S2

## Additional Information and Declarations

### Data Availability

The following information was supplied regarding data availability: Figshare: https://doi.org/10.6084/m9.figshare.5116759.
