# Supplementary material for: Genus-level phylogeny of cephalopods using molecular markers: current status and problematic areas
Source: PeerJ. 2018 Feb 12;6:e4331. doi: 10.7717/peerj.4331 (PMC5813590; doi:10.7717/peerj.4331)
Supplement: Supplemental Information 1 [file peerj-06-4331-s001.docx]

**Supplementary Table 1:** Accession numbers retrieved from GenBank and the Barcode of Life Database, used in the present study.

| **Genus** | **12S** | **16S** | **18S** | **Actin** | **COI** | **Histone** | **ODH** | **Pax6** | **Rhodopsin** | **Full Mitogenome** |
| --- | --- | --- | --- | --- | --- | --- | --- | --- | --- | --- |
| *Nautilus* | AY616965.1 | AY377628.1 | AY145384.1 | AF234901.1 | NC_007980.1 |  |  | AY617039.1 |  |  |
| *Allonautilus* |  | GQ280184.1 |  |  | KP892752.1 |  |  |  |  |  |
| *Spirula* | AY293631.1 | X79574.1 | AY557476.1 | AF234914.1 | JQ412176.1 | AY557420.1 |  | AY545159.1 | AY545183.1 |  |
| *Sepioteuthis* | AB699275.1 | KF052137.1 | AY557480.1 | AF234924.1 | AB240154.1 | AY557424.1 | KF052503.1 | AY616932.1 | AY616917.1 | AB240154.1 |
| *Lolliguncula* | AY616867.1 | AF110085.2 | EU735268.1 |  | EU735357.1 | EU735405.1 | AY616900.1 | AY616931.1 | AY616916.1 |  |
| *Doryteuthis* | NC_012840.1 | KF854019.1 |  |  | NC_012840.1 |  |  |  |  | NC_012840.1 |
| *Heterololigo* | AB699305.1 | AB191142.1 |  |  | NC_002507.1 |  |  |  |  | NC_002507.1 |
| *Afrololigo* |  |  |  |  | EU668101.1 |  |  |  | EU668060.1 |  |
| *Alloteuthis* |  | EU668106.1 |  |  | KM517865.1 |  |  |  | EU668049.1 |  |
| *Loliolus* | AB699312.1 | HQ529547.1 |  |  | KP265013.1 |  | AY616904.1 | AY616935.1 | AY616920.1 | KP265013.1 |
| *Uroteuthis* | AB675080.1 | KF032034.1 |  |  | AB675080.1 |  |  |  |  | AB675080.1 |
| *Loligo* | AB699272.1 | AY185505.1 | AF308647.1 | AY701849.1 | KM517903.1 | AY377782.1 | AJ250884.1 | AY545161.1 | AY545184.1 |  |
| *Idiosepius* | AB191148.1 | AY293658.1 | AY557477.1 | AB500119.1 | AF000046.1 | AY557421.1 |  | AB716344.1 | AY545181.1 |  |
| *Alluroteuthis* |  |  |  | AF234954.1 | AF131871.1 |  |  |  |  |  |
| *Chtenopteryx* | AY293630.1 | HQ613156.1 | EU735270.1 | AF234931.1 | HQ386018.1 | EU735407.1 |  |  |  |  |
| *Abralia* |  | HQ845987.1 | EU735279.1 | AF234941.1 | AF000025.1 | EU735417.1 |  |  |  |  |
| *Thysanoteuthis* | AB699284.1 | EU735236.1 | EU735301.1 | AF234965.1 | GU188438.1 | EU735447.1 |  |  |  |  |
| *Asperoteuthis* | EU421720.1 | EU421719.1 | EU735306.1 |  | EU421718.1 |  |  |  |  |  |
| *Grimalditeuthis* |  | EU735226.1 | EU735285.1 |  | EU735363.1 | EU735430.1 |  |  |  |  |
| *Chiroteuthis* | KC861168.1 | EU735246.1 | EU735284.1 | AF234929.1 | AF000032.1 | EU735429.1 |  |  |  |  |
| *Planctoteuthis* |  | EU735247.1 | EU735281.1 |  | EU735384.1 | EU735431.1 |  |  |  |  |
| *Joubiniteuthis* | AY616865.1 | EU201153.1 | AY557502.1 |  | AF000048.1 | AY557444.1 | AY616897.1 | AY545159.1 | AY616914.1 |  |
| *Brachioteuthis* |  | EU735201.1 | AY557485.1 | AF234928.1 | KF644162.1 | EU735411.1 |  |  |  |  |
| *Neoteuthis* |  | EU735215.1 | AY557506.1 |  |  | AY557448.1 |  |  |  |  |
| *Magnapinna* |  | EU735227.1 | EU735302.1 |  | EU735364.1 | EU735440.1 |  |  |  |  |
| *Mastigoteuthis* | EU201149.1 | EU201159.1 | AY557505.1 | AF234953.1 | KC860971.1 | EU735432.1 |  |  |  |  |
| *Batoteuthis* |  | EU735200.1 | AY557484.1 |  |  | AY557428.1 |  |  |  |  |
| *Histioteuthis* | AY616954.1 | DQ280047.1 | AY145378.1 | AF234950.1 | GU220787.1 | EU735425.1 | AY616999.1 | AY617028.1 | AY617053.1 |  |
| *Architeuthis* | NC_011581.1 | HQ638200.1 | AY557482.1 |  | NC_011581.1 | EU735406.1 | AY617007.1 |  | AY617052.1 | NC_011581.1 |
| *Psychroteuthis* |  | EU735221.1 | AY557513.1 | AF234963.1 | AF131876.1 | AY557454.1 |  |  |  |  |
| *Leachia* |  | EU735252.1 | EU735275.1 |  | GU145067.1 | AY557431.1 |  |  |  |  |
| *Cranchia* | AY616962.1 | DQ280046.1 | AY557487.1 | AF234935.1 | GQ853498.1 | AY557430.1 | AY617015.1 | AY617029.1 | AY617061.1 |  |
| *Abraliopsis* | AY616955.1 | X79595.1 | AY145364.1 |  |  | AY557434.1 |  | AY617029.1 | AY617054.1 |  |
| *Enoploteuthis* | AY616956.1 | AJ223485.1 | AY557493.1 | AF234944.1 | AF000039.1 | AY557435.1 | AY617003.1 | AY617030.1 | AY617055.1 |  |
| *Discoteuthis* |  | EU735205.1 | AY557490.1 | AF234940.1 | AF000037.1 | AY557433.1 |  |  |  |  |
| *Cycloteuthis* |  | EU735204.1 | AY557489.1 | AF234938.1 | AF000036.1 | AY557432.1 |  |  |  |  |
| *Onychoteuthis* | AY616968.1 | EU735250.1 | EU735296.1 | AF234960.1 | GQ853500.1 | EU735446.1 | AY616998.1 |  | AY617051.1 |  |
| *Ancistroteuthis* |  | EU735234.1 | EU735300.1 |  | EU735379.1 | EU735443.1 |  |  |  |  |
| *Notonykia* |  | EU735232.1 | EU735298.1 |  | EU735378.1 | EU735444.1 |  |  |  |  |
| *Onykia* |  | AJ223489.1 | EU735297.1 |  | GU112107.1 | EU735442.1 |  |  |  |  |
| *Kondakovia* |  | EU735267.1 | EU735314.1 |  | EU735403.1 | EU735445.1 |  |  |  |  |
| *Liocranchia* |  | X79587.1 |  | AF234936.1 | AF000050.1 |  |  |  |  |  |
| *Taonius* |  | EU735245.1 | EU735276.1 |  | EU735381.1 | EU735413.1 |  |  |  |  |
| *Teuthowenia* | AY616961.1 | AY616984.1 |  |  | AY617064.1 |  | AY617010.1 | AY617035.1 | AY617060.1 |  |
| *Mesonychoteuthis* |  | EU735261.1 | EU735308.1 |  | EU735397.1 | EU735416.1 |  |  |  |  |
| *Helicocranchia* |  | AF110099.2 |  |  | GU145078.1 |  |  |  |  |  |
| *Galiteuthis* | AY616959.1 | AY616987.1 | EU735307.1 |  | KF309247.1 | EU735415.1 | AY616997.1 | AY617029.1 | AY617058.1 |  |
| *Megalocranchia* | AY616960.1 | EU735228.1 | EU735277.1 |  | EU735382.1 | EU735412.1 | AY617001.1 | AY617029.1 | AY617059.1 |  |
| *Bathyteuthis* | AY616863.1 | EU735222.1 | AY557483.1 | AF234925.1 | AP012225.1 | EU735409.1 | AY617002.1 | AY616927.1 | AY616912.1 | AP012225.1 |
| *Todaropsis* | AY616964.1 | AY616988.1 | KJ193806.1 |  | KM517968.1 |  | AY617000.1 | AY617038.1 | AY617063.1 |  |
| *Octopoteuthis* | AY616957.1 | EU735258.1 | EU735292.1 | AF234955.1 | KM612266.1 | EU735437.1 | AY617013.1 | AY617031.1 | AY617056.1 |  |
| *Lepidoteuthis* |  | DQ280048.1 | AY557503.1 | AF234951.1 | AF000049.1 | AY557445.1 |  |  |  |  |
| *Lycoteuthis* |  | EU735257.1 | EU735304.1 |  | AF131874.1 | EU735420.1 |  |  |  |  |
| *Selenoteuthis* |  | EU735230.1 | EU735303.1 |  | EU735367.1 |  |  |  |  |  |
| *Ancistrocheirus* |  |  | AY557491.1 |  | GU220396.1 |  |  |  |  |  |
| *Pyroteuthis* |  | EU735209.1 | AY557496.1 | AF234964.1 | AF000060.1 | AY557438.1 |  |  |  |  |
| *Pterygioteuthis* | AY616864.1 | EU735253.1 | EU735280.1 |  | JQ248117.1 | EU735419.1 | AY616895.1 | AY616928.1 | AY616913.1 |  |
| *Eucleoteuthis* | AB699285.1 | EU735244.1 | EU735294.1 |  | AB270945.1 | EU735434.1 |  |  |  |  |
| *Hyaloteuthis* |  | AB270962.1 |  |  | AB270947.1 |  |  |  |  |  |
| *Sthenoteuthis* | AB699286.1 | EU658923.1 | AY557511.1 | AF234959.1 | AB270943.1 | AY557452.1 | AY617005.1 | AY545162.1 | AY545185.1 | NC_010636.1 |
| *Ommastrephes* | AB699274.1 | AB509422.1 | AY557510.1 | AF234957.1 | AB270941.1 | AY557451.1 | AY616899.1 | AY545162.1 | AY616915.1 | AB715401.1 |
| *Dosidicus* | AB699297.1 | NC_009734.1 | EU735293.1 |  | FJ153074.1 | EU735436.1 |  |  |  | NC_009734.1 |
| *Illex* | AB699279.1 | X79594.1 | AY557509.1 |  | KP336702.1 | AY557450.1 | AY617008.1 | AY617037.1 | AY617062.1 | KP336702.1 |
| *Nototodarus* |  | GQ365324.1 |  |  | AB270937.1 |  |  |  |  |  |
| *Ornithoteuthis* |  | EU735207.1 | AY557494.1 |  | AB270946.1 | AY557436.1 |  |  |  |  |
| *Martialia* |  | AB270955.1 |  |  | AB270940.1 |  |  |  |  |  |
| *Todarodes* | AB699295.1 | X79589.1 |  | FJ611946.1 | AB270935.1 |  |  |  |  | AB158364.1 |
| *Pholidoteuthis* |  | EU735254.1 | EU735305.1 | AF234962.1 | EU735390.1 | EU735448.1 |  |  |  |  |
| *Berryteuthis* | AB191147.1 | AY681048.1 | EU735310.1 |  | GQ856242.1 | EU735423.1 |  |  |  |  |
| *Gonatus* | AY681006.1 | AY681034.1 | AY557498.1 | AF234945.1 | GU072734.1 | AY557439.1 |  |  |  |  |
| *Gonatopsis* | AY680997.1 | AY681031.1 | EU735311.1 | AF234947.1 | AB749275.1 | EU735421.1 |  |  |  |  |
| *Watasenia* | AB086202.1 | AB191139.1 |  |  | KJ845633.1 |  |  |  |  | AB240152.1 |
| *Heteroteuthis* | AY616873.1 | AY293680.1 | AY557472.1 | AF234910.1 | AB591074.1 | AY557416.1 | AY616906.1 | AY545158.1 | AY616922.1 |  |
| *Rossia* | AB191149.1 | AB191138.1 | AY557473.1 | AF234911.1 | KM517929.1 | AY557417.1 | AY616905.1 | AY616936.1 | AY616921.1 |  |
| *Sepiola* | AY293642.1 | KR259947.1 | AY557474.1 |  | KM517949.1 | AY557418.1 |  | AY545158.1 | AY545182.1 |  |
| *Sepietta* | AY293647.1 | AY293674.1 | AJ606937.1 |  | KM517942.1 |  |  |  |  |  |
| *Euprymna* | AB191151.1 | AY293665.1 | AY149446.1 | AY149451.1 | AF350493.1 |  | AY616907.1 | AF513712.1 | EU344773.1 |  |
| *Rondeletiola* | AY293651.1 | AY293677.1 |  |  | AF035714.1 |  |  |  |  |  |
| *Sepiolina* | AY293653.1 | AY293679.1 |  |  | AB591071.1 |  |  |  |  |  |
| *Sepia* | AB699276.1 | AB675086.1 |  |  | NC_022466.1 |  |  | AY617024.1 | AY617047.1 |  |
| *Metasepia* | AB192333.1 | AB192340.1 |  |  | HQ846120.1 |  | AY616909.1 | AY616940.1 | AY616925.1 |  |
| *Sepiella* | AB192334.1 | AB192341.1 | AY557470.1 | JN564496.1 | KF040369.1 | EU735404.1 | AY616996.1 | AY545160.1 | AY617048.1 | KF040369.1 |
| *Sepiadarium* | AB191152.1 | AY293678.1 |  |  | AY293726.1 |  | AY616995.1 | AY617023.1 | AY617046.1 |  |
| *Sepioloidea* | AY616948.1 | AY616975.1 |  | AF234916.1 | AF000064.1 |  | AY616994.1 | AY617022.1 | AY617045.1 |  |
| *Vampyroteuthis* | AY545077.1 | DQ280043.1 | AY145387.1 | AF234981.1 | AB266515.1 | AY557408.1 |  | AY545139.1 | AY545163.1 | AB266515.1 |
| *Cirroteuthis* |  | AF487284.1 |  |  |  |  |  |  |  |  |
| *Cirrothauma* |  | AF487282.1 | AY557456.1 | AF234968.1 | AF000034.1 |  |  |  |  |  |
| *Stauroteuthis* | AY545078.1 | DQ280042.1 | AY557457.1 | AF234969.1 | AF000067.1 | AY557406.1 |  | AY545140.1 | AY545164.1 |  |
| *Luteuthis* |  | AJ315377.1 |  |  |  |  |  |  |  |  |
| *Opisthoteuthis* | AY545079.1 | AJ315372.1 | AY557458.1 |  | AF377961.1 | AY557407.1 |  | AY545141.1 | HM104301.1 |  |
| *Grimpoteuthis* |  | AF110100.2 |  |  | AF377963.1 |  |  |  |  |  |
| *Cirroctopus* |  | AJ315376.1 |  |  | GU073528.1 |  |  |  |  |  |
| *Tremoctopus* | AY545081.1 | AJ252767.1 |  |  | AF377978.1 |  |  | AY545143.1 | AY545167.1 |  |
| *Haliphron* | AY616942.1 | AY616971.1 | AY557460.1 |  | AF377964.1 | AY557410.1 | AY616910.1 | AY617016.1 | AY617040.1 |  |
| *Cistopus* | KF017605.1 | AJ252744.1 |  |  | KF017605.1 |  | JR436137.1 | HM104271.1 | AY617044.1 | KF017605.1 |
| *Ameloctopus* | HM104235.1 | HM104245.1 |  |  | HM104255.1 |  | HM104277.1 | HM104266.1 | HM104288.1 |  |
| *Hapalochlaena* | AB191127.1 | GQ900711.1 |  |  | JN790685.1 |  |  | AY545147.1 | AY545171.1 |  |
| *Amphioctopus* | AB191125.1 | KF589848.1 |  |  | JN790684.1 |  |  | HM104267.1 | HM104289.1 | NC_007896.1 |
| *Octopus* | AB191119.1 | GU362545.1 | FJ617439.1 | FJ611947.1 | AB158363.1 |  | HM104284.1 | HM104274.1 | KR902901.1 |  |
| *Abdopus* | HM104234.1 | GQ900716.1 |  |  | AB430514.1 |  |  | HM104265.1 | HM104287.1 |  |
| *Thaumoctopus* |  | GQ900725.1 |  |  | GQ900746.1 |  |  |  |  |  |
| *Wunderpus* |  | GQ900723.1 |  |  | GQ900748.1 |  |  |  |  |  |
| *Callistoctopus* | AB191128.1 | GQ900705.1 |  |  | HQ638215.1 |  |  |  |  | HQ638215.1 |
| *Robsonella* |  | KC792310.1 |  |  |  |  |  |  |  |  |
| *Scaeurgus* | HM104242.1 | HM104248.1 |  |  | HM104263.1 |  | HM104285.1 |  | HM104298.1 |  |
| *Grimpella* | HM104238.1 | HM104246.1 |  |  | HM104259.1 |  | HM104282.1 | HM104272.1 | HM104295.1 |  |
| *Velodona* | EU071440.1 | EU071434.1 |  |  |  |  |  | EU071419.1 | HM104299.1 |  |
| *Thaumeledone* | EU071439.1 | EU071432.1 | AY557469.1 |  | GU073559.1 | AY557414.1 |  | EU071409.1 | EU086514.1 |  |
| *Ocythoe* | GU288528.1 | GU288520.1 | AY557464.1 |  |  |  |  |  |  |  |
| *Argonauta* | GU288526.1 | AB191108.1 | AY557462.1 | AF234970.1 | AB191273.1 | AY557411.1 |  | AY545142.1 | AY545166.1 |  |
| *Enteroctopus* | AB191121.1 | HM572165.1 |  |  | GU802397.1 |  |  | AY545150.1 | AY545174.1 |  |
| *Muusoctopus* | FJ603548.1 | KM459464.1 | AY145366.1 |  | GU073624.1 | AY557412.1 | AY616991.1 | HM104270.1 | AY545173.1 |  |
| *Sasakiopus* | GQ226029.1 | GQ226031.1 |  |  |  |  |  |  | GQ226025.1 |  |
| *Bathypolypus* | EF016348.1 | DQ280044.1 | AY557465.1 | AF234977.1 | AF377969.1 |  | HM104279.1 | AY617017.1 | GQ226024.1 |  |
| *Bentheledone* |  | AJ311117.1 |  |  | AF377975.1 |  |  |  |  |  |
| *Graneledone* | AY545091.1 | JN800402.1 | AY145376.1 | AF234978.1 | AF377974.1 | AY557413.1 | HM104281.1 | HM104273.1 | EU086516.1 |  |
| *Megaleledone* | EF102216.1 | EF102195.1 |  |  | GU073512.1 |  |  | EU071420.1 | EF102114.2 |  |
| *Pareledone* | EF102235.1 | EF102210.1 |  |  | GU073490.1 |  |  | AY545152.1 | AY545176.1 |  |
| *Adelieledone* | EF102215.1 | EU071431.1 |  |  | GU073471.1 |  |  | AY545151.1 | AY545175.1 |  |
| *Amphitretus* |  |  |  |  | AF377965.1 |  |  |  |  |  |
| *Vitreledonella* | AY545092.1 | AY545112.1 |  |  | AF000072.2 |  |  | AY545154.1 | AY545178.1 |  |
| *Bolitaena* | AY545094.1 |  |  |  |  |  |  | AY545156.1 | AY545180.1 |  |
| *Japetella* | AY545093.1 | AJ252766.1 | AY557463.1 | AF234974.1 |  |  |  | AY545155.1 | AY545179.1 |  |
| *Eledone* | AY616946.1 | AJ252764.1 | AY557467.1 |  | KM517894.1 |  | AY616992.1 | AY617020.1 | HM104292.1 |  |

**Supplementary Table 2:** Corrected names for misplaced or misidentified taxon in databases

| **Database taxon** | **Assigned taxon** |
| --- | --- |
|  |  |
| *Moroteuthis knipovitchi* | Filippovia |
|  |  |
| Moroteuthis | Onykia |
|  |  |
| Benthoctopus | Muusoctopus |
|  |  |
| Vulcanoctopus | Muusoctopus |
|  |  |
| Praealtus | Graneledone |
|  |  |
| Tetracheledone | Graneledone |
|  |  |
| *Octopus aegina* | Amphioctopus |
|  |  |
| *Octopus ornatus* | Callistoctopus |
|  |  |
